# Assessment of Potential Live Kidney Donors and Computed Tomographic Renal Angiograms at Christchurch Hospital

**DOI:** 10.1155/2016/4924320

**Published:** 2016-03-10

**Authors:** Thamer Alsulaiman, Stephen Mark, Sarah Armstrong, David McGregor

**Affiliations:** Christchurch Hospital, Private Bag 4710, Christchurch 8140, New Zealand

## Abstract

*Aims*. To examine the outcome of potential live kidney donors (PLKD) assessment program at Christchurch Hospital and, also, to review findings of Computed Tomographic (CT) renal angiograms that led to exclusion in the surgical assessment.* Methods*. Clinical data was obtained from the database of kidney transplants, Proton. Radiological investigations were reviewed using the hospital database, Éclair. The transplant coordinator was interviewed to clarify information about PLKD who did not proceed to surgery, and a consultant radiologist was interviewed to explain unfavorable findings on CT renal angiograms.* Results*. 162 PLKD were identified during the period January 04–June 08. Of those, 65 (40%) proceeded to have nephrectomy, 15 were accepted and planned to proceed to surgery, 13 were awaiting further assessment, and 69 (42.5%) did not proceed to nephrectomy. Of the 162 PLKD, 142 (88%) were directed donors. The proportion of altruistic PLKD who opted out was significantly higher than that of directed PLKD (45% versus 7%, *P* = 0.00004).* Conclusions*. This audit demonstrated a positive experience of live kidney donation at Christchurch Hospital. CT renal angiogram can potentially detect incidental or controversial pathologies in the kidney and the surrounding structures. Altruistic donation remains controversial with higher rates of opting out.

## 1. Introduction

Kidney transplantation has been widely accepted as the treatment of choice for end stage renal disease [1]. Live kidney donation has been one strategy to increase the number of kidney grafts available. Living donation offers many advantages for the kidney recipient, including a reduced waiting time for transplant and a significantly reduced ischemic (storage) time [1, 2]. Two forms of nephrectomies have been historically utilized to harvest the kidney: open and laparoscopic. The first laparoscopic live donor kidney nephrectomy performed in New Zealand was in Auckland, June 2000, after being first performed in Baltimore, 1995 [1]. Since then, the number of live donor nephrectomies has increased in New Zealand [1]. The laparoscopic approach has become the standard approach for harvesting kidneys from live donors, with the left kidney being preferred by most surgeons because of possessing a longer vein and being technically easier to remove [3]. Laparoscopic nephrectomy has many advantages compared with open nephrectomy. These include decreased estimated blood loss intraoperatively, decreased pain and cost of hospital stay postoperatively, and shorter interval before resumption of work and full activities [4–6].

Previously, a conventional renal angiogram was the investigation of choice used to look at renal vessels to assess live donors surgically. Currently, a spiral CT renal angiogram is being utilized. The CT renal angiogram offers detailed anatomy of renal vessels, renal parenchyma, and collecting ducts. It also offers details about surrounding abdominal structures. This audit looked at general trends and outcomes of the PLKD assessment program at Christchurch Hospital. It also looked in particular at the outcome of CT renal angiogram utilized in the surgical assessment.

## 2. Patients and Methods

Christchurch Hospital provides transplant services for approximately a quarter of New Zealand population (one million in the South Island). The assessment process for live kidney donors in Christchurch involves a stepwise series of tests and consultations and can take three to nine months after the initial contact with the transplant coordinator by voluntary donors. PLKD expressing interest in the assessment that are ABO blood group compatible meet with a donor counselor. A psychological review is conducted for altruistic (nondirected) PLKD. For candidates who wish to proceed, a tissue type crossmatching test is performed and a routine medical assessment is then done by a nephrologist. Finally, PLKD undergo surgical assessment. The medical assessment involves taking a history, examination, and a series of blood and functional tests assessing respiratory, cardiac, and renal systems. Blood tests involve viral studies (HIV, Hepatitis B, Hepatitis C, CMV, and EBV), full blood count, electrolytes, liver enzymes, lipids, and coagulation profile. Routine investigations include chest X-ray, ECG, and renal and bladder ultrasound scan (USS). Glomerular filtration rate (GFR) is estimated using a radionuclide scan. Obese donors (BMI > 30) are generally excluded during medical assessment. Once medical assessment is complete, PLKD proceed to surgical assessment, which involves a spiral CT renal angiogram followed by an appointment with the surgeon for counseling and discussion of assessment results. PLKD can opt out of the assessment program at any stage for any personal reason or simply because of a change of heart.

In this study, the database of kidney transplants, Proton, at the department of urology and nephrology was accessed. The search included any patient in the Proton database that was classified as “donor” and had his “first biochemistry test” during the period “January 04–June 08” (54 months). The database provided information on whether PLKD proceeded or not to have nephrectomy. It also provided information about the type of nephrectomy that was planned or performed. The Éclair hospital database was used to provide information on the type of radiological investigations each PLKD had (renal USS, renal angiogram, and CT renal angiogram). Reports on the results of these investigations were reviewed and abnormal findings were noted. PLKD who did not proceed to have a nephrectomy were further investigated for the reason and categorized accordingly. This was done by looking at information provided in the Proton database, reviewing reports of radiological investigations, and interviewing the transplant coordinator. In this audit, PLKD who did not proceed to have nephrectomy based on CT renal angiogram findings were a group of interest. Reasons for being declined based on CT renal angiograms were identified through the radiologist report on the Éclair database and discussed with a consultant radiologist. Information on “whether the donation was directed or not” was obtained from the Proton database. Directed donations were identified as those where the donor specified the recipient for his kidney. These included blood relatives, spouses, and friends. Nondirected donations were identified as those where the donor identified himself/herself as an altruistic donor.

## 3. Results

During the period January 04–June 08, 162 PLKD were identified. 80 PLKD (49.5%) fulfilled the assessment criteria. Of these, 48 proceeded to have a laparoscopic nephrectomy, 17 proceeded to have an open nephrectomy, and 15 were awaiting their operation to be planned. 13 PLKD were awaiting further assessment and 69 (42.5%) did not proceed to have a nephrectomy. Of the 69 PLKD who did not proceed to nephrectomy, 19 opted out at different stages of the assessment program, 17 were declined on medical grounds, 17 did not proceed because of a change in the recipient situation (e.g., recipient getting a kidney off the transplant list or becoming medically unwell and unfit for the transplant operation), 11 were declined based on the findings of CT renal angiogram, 4 had incompatible tissue type crossmatch, and 1 was declined based on the renal USS finding of bilateral renal cysts.

93 CT renal angiograms were performed during the period January 04–June 08 as part of the live kidney donation assessment program in Christchurch. Of these, 11 (12%) had unfavorable findings leading them to be declined for surgery, Table 1. Among PLKD who had an acceptable CT renal angiogram, 2 opted out of the program while 3 became medically unfit closer to the time of operation (e.g., one had a hypertensive crisis on the day of operation).

142 (88%) of the donations were directed. Of the 19 PLKD who opted out at different stages of the assessment program, 9 were nondirected and 10 were directed donors. The proportion of nondirected PLKD who opted out was significantly higher than that of directed PLKD (45% versus 7%, *P* = 0.00004), Table 2.

## 4. Discussion

### 4.1. Outcome of Assessment of Potential Live Kidney Donors

This audit demonstrated a positive experience of live kidney donation at Christchurch Hospital. It also provided some baseline data about PLKD undergoing assessment during January 04–June 08. The proportion of PLKD proceeding to nephrectomy in Christchurch (40%) compares well to other centers. The number of laparoscopic nephrectomies was three times that of open nephrectomies (60 versus 19). The type of nephrectomy was mainly determined by the surgeon's preference, vascular anatomy, and anticipated complexity of procedure. McCurdie et al. reported on the outcome of assessment of 117 PLKD at the transplant unit, Cape Town, South Africa, that were evaluated over a 39-month period (January 2000–March 2003). A very similar stepwise progression of counseling and testing was described in their report with CT renal angiogram being the investigation of choice for surgical assessment. They found that only 17% of PLKD assessed were eventually used, 61% were found unsuitable for reasons ranging from blood group incompatibility to serious medical conditions, and 22% did not proceed because of change in recipient situation [7].

In this paper, the most common reason for not proceeding to nephrectomy was “donor opting out (27.5%).” This figure reflects the flexibility of the PLKD assessment program in Christchurch in respecting the PLKD wishes to continue or to opt out of the program at any stage. Because the assessment process can be lengthy and because every PLKD is offered a number of counseling sessions (with a donor counselor, psychiatrist, physician, and surgeon), PLKD have the opportunity to think and take the time to understand the procedure of kidney transplant and risks involved which is essential from an ethical point of view to make an informed consent. Although an increasing number of PLKD withdrawing from the program especially at advanced stages of assessment (e.g., two PLKD opted out after having normal CT renal angiograms in this paper) can be time-consuming and not cost-effective, the number of PLKD proceeding to surgery after a comprehensive assessment and counseling is worthwhile (40%) and might be associated with better outcomes in terms of donor expectations and satisfaction after the operation. An attempt was made to explain the number of PLKD who opted out since specific reasons for opting out were not necessarily required by donor or documented in the Proton database. PLKD were classified into directed or nondirected (altruistic) donors to test the hypothesis that those who opted out are more likely to be nondirected donors. Figures in this paper indicate that being a nondirected donor was significantly associated with opting out (45% versus 7%, *P* = 0.00004), Table 2.

The second most common two reasons were “change in recipient situation (24.6%)” and finding the donor “medically unfit (24.6%)” during the medical assessment. Recipients on the transplant waiting list may become unwell while awaiting their operation or find an alternative donor. This change in recipient situation cannot be easily controlled by a transplant center as a reason for PLKD not proceeding to surgery. Medical reasons ranged from reduced lung function, reduced kidney function, hypertensive crisis, and obesity to diagnosing a cancer (prostate) in one of the donors. Being declined because of unfavorable findings on CT renal angiogram was the third most common reason (16%). Different transplant centers have different exclusion criteria regarding surgical assessment on CT renal angiogram and anticipated degree of complexity of the transplant operation. For example, in Christchurch, a unilateral simple cyst in a kidney would not be a contraindication to having the procedure. This is not the case in other centers, such as Auckland. Angiomyolipoma of the kidney is another controversial finding, especially if small, asymptomatic, and proved to be benign. Three PLKD in this paper were declined because of findings of markers for developing vascular abnormalities (aneurysm in the common iliac artery, mesenteric lymphadenopathy, and occluded celiac axis) thus making the decision of leaving them with one kidney unfavorable. Six PLKD that proceeded to nephrectomy had incidental findings on CT renal angiogram (four lesions in the liver (hemangioma), one chest nodule, and one adrenal adenoma). Those PLKD had to undergo further waiting and investigations before deciding on the significance of the incidental findings. One PLKD was declined because of bilateral renal cysts on CT renal angiogram. Interestingly, only one cyst was identified on the renal USS.

Having incompatible tissue type crossmatching (0.04%) and an abnormal USS (0.01%) were other reasons not to proceed to nephrectomy. The abnormal finding on the USS was bilateral renal cysts, which carry a risk of progression of cyst pathology in the donor or recipient's kidney. The number of PLKD with incompatible crossmatch is probably underestimated in this paper because this group would not have proceeded to have a biochemistry test as part of the medical assessment and thus was not expected to be picked up in the search. However, four PLKD who were crossmatch incompatible were picked up by chance. It is also worthwhile to mention that PLKD who were ABO incompatible did not proceed to further assessment and were not classified as “donors” in the Proton database. Therefore, no information was found about them.

### 4.2. CT Renal Angiography of Living Renal Donors

Assessment of PLKD before proceeding to nephrectomy requires careful evaluation of renal arterial and venous anatomy. CT angiography is a minimally invasive test that has been an accepted method for preoperative evaluation of potential renal donors [3, 8]. CT renal angiogram has the advantage of being noninvasive compared with conventional renal angiogram. It only requires an antecubital venous access and can be done as an outpatient procedure with the patient being ambulatory immediately after the procedure. In contrast, conventional renal angiography is invasive and 6–8 hours of bed rest is mandatory [9]. Disadvantages of the CT renal angiogram include a theoretical risk of contrast-induced renal failure, which is rarely encountered in normally functioning kidneys [9].

Unfavorable findings on CT renal angiogram leading to exclusion from donor nephrectomy include anatomical, vascular, and collecting duct abnormalities. Renal anomalies (e.g., horseshoe kidney), renal neoplasm, polycystic kidney disease, and cortical atrophy are identified reasons for exclusion from donor nephrectomy [3, 10, 11]. Nephrolithiasis is a relative contraindication because of the risk of recurrence in recipients and recurrence with injury to the donor while unilateral small cysts are usually not a contraindication [3]. Angiomyolipoma and hemangioma are forms of neoplasm that can be incidentally encountered on the CT renal angiogram. The significance of the neoplasm is assessed by a noncontrast CT to evaluate the degree of enhancement. Vascular lesions such as stenosis secondary to fibromuscular dysplasia are a reason for exclusion from nephrectomy that can be well evaluated on a CT renal angiogram [3, 10]. Other lesions include aneurysms and arteriovenous malformation, which are markers for vascular abnormalities. 75% of individuals will have a single renal artery bilaterally while bilateral single renal veins are found in 92% [3]. Anatomic variants of the left renal vein such as circumaortic and retroaortic renal veins can add complexity to the nephrectomy procedure and need to be identified preoperatively. However, these variations are not usually a contraindication [3, 10]. A good evaluation of the collecting system and ureters is provided by the CT renal angiogram. Duplication of the renal collecting system, hydronephrosis, uroepithelial tumours, and ureteropelvic junction obstruction can be incidental findings that affect the surgical planning [3].

Previous studies have demonstrated that spiral CT angiography can replace intravenous urography and renal angiography, which were traditionally used for evaluation of potential renal donors [3]. Moreover, it has been reported to be as accurate as renal angiography for arterial anatomy and more sensitive than renal angiography and intravenous urography in evaluation of the venous and parenchymal anatomy [9, 12, 13]. Furthermore, the use of CT angiography instead of excretory urography and conventional angiography can result in 35–50% reduction of cost in the imaging studies of PLKD [12].

## 5. Conclusions

Figures in this audit demonstrated a positive experience in the live kidney donation program at Christchurch Hospital. CT renal angiogram is a useful tool in the surgical evaluation of potential live kidney donors. Its use has been reported to be accurate and more cost-effective than plain renal angiogram. Although CT renal angiogram can potentially detect incidental or controversial pathologies in the kidney and surrounding structures, the relevance of these pathologies is determined by the transplant surgeons and radiologist involved in the surgical assessment. Different transplant centers will have different protocols and exclusion criteria for undergoing a nephrectomy based on experience, operating surgeons' preference, and the donors' willingness to undertake certain risks. Being an altruistic donor was found to be associated with higher rates of opting out. The significance of this finding may have different implications in different transplant centers depending on their views regarding the controversy of altruistic donation.

## Figures and Tables

**Table 1:** Unfavorable findings on CT renal angiograms.

1	Kidney scarring
2	Fibromuscular dysplasia of renal artery
3	Arteriovenous malformation
4	Complex renal vasculature
5	Bilateral renal cysts
6	Right common iliac artery aneurysm
7	Mesenteric lymphadenopathy
8	Angiomyolipoma
9	Complex renal vasculature
10	Occluded celiac axis
11	Nephrolithiasis

**Table 2:** Directed versus nondirected PLKD.

PLKD	Directed	Nondirected	Total
Did not opt out	132	11	143
Opted out	10	9	19
Total	142	20	162
